# Short-term culture of tumour slices reveals the heterogeneous sensitivity of human head and neck squamous cell carcinoma to targeted therapies

**DOI:** 10.1186/s12885-016-2318-x

**Published:** 2016-04-16

**Authors:** Jérôme Donnadieu, Emma Lachaier, Marine Peria, Zuzana Saidak, Stéphanie Dakpe, Jean-Fortune Ikoli, Bruno Chauffert, Cyril Page, Antoine Galmiche

**Affiliations:** Department of Head and Neck Surgery, University Hospital, Amiens, France; Department of Medical Oncology, University Hospital, Amiens, France; Department of Biochemistry, University Hospital, Amiens, France; Department of Maxillofacial Surgery, University Hospital, Amiens, France; Department of Pathology, University Hospital, Amiens, France; EA4666, Université de Picardie-Jules Verne (UPJV), Amiens, France

**Keywords:** Head and neck squamous cell carcinoma, Short-term culture of tumour fragments, Targeted therapies, Treatment personalization

## Abstract

**Background:**

Despite recent progress, investigating the impact of targeted therapies on Head and Neck Squamous Cell Carcinoma (HNSCC) remains a challenge. We investigated whether short-term culture of tumour fragments would permit the evaluation of tumour sensitivity to targeted therapies at the individual level.

**Methods:**

We cultivated tumour slices prepared from 18 HNSCC tumour samples obtained during surgical resection. The samples were treated for 48 h with a panel of 8 targeted therapies directed against selected oncogenic transduction pathways. We analysed the cell proliferation index (CPI) of tumour cells using Ki67 labelling and the activation status of the RAF-MEK-ERK cascade through ERK phosphorylation analysis.

**Results:**

Fourteen tumours were successfully analysed after short-term culture of tumour samples, revealing a striking individual heterogeneity of HNSCC in terms of tumour cell sensitivity to targeted therapies. Using 50 % inhibition of CPI as threshold, sorafenib was shown to be active in 5/14 tumours. Cetuximab, the only approved targeted drug against HNSCC, was active in only 2/14 tumours. A more than 50 % inhibition was observed with at least one drug out of the eight tested in 10/14 tumours. Cluster analysis was carried out in order to examine the effect of the drugs on cell proliferation and the RAF-MEK-ERK cascade.

**Conclusions:**

In vitro culture of tumour fragments allows for the evaluation of the effects of targeted therapies on freshly resected human tumours, and might be of value as a possible guide for the design of clinical trials and for the personalization of the medical treatment of HNSCC.

**Electronic supplementary material:**

The online version of this article (doi:10.1186/s12885-016-2318-x) contains supplementary material, which is available to authorized users.

## Background

Head and neck squamous cell carcinomas (HNSCC) are tumours characterized by great phenotypic, aetiological and biological heterogeneity among individuals [1]. Standard options for locally advanced HNSCC are surgery and adjuvant radiotherapy or cisplatin-based chemoradiotherapy, depending on associated risk factors [2]. Chemoradiotherapy is used alone for non-resectable tumours [2]. However, a large number of patients display locoregional and/or metastatic recurrence despite an adequate local treatment. Cetuximab, a monoclonal antibody directed against the Epidermal Growth Factor Receptor (EGFR), is the only targeted therapy approved for advanced HSNCC. It is used in combination with radiotherapy for locally advanced disease [3] or with platinum-based chemotherapy for palliative purposes [4, 5]. Epidermal Growth Factor (EGFR) is almost systematically overexpressed in HNSCC, but no clear correlation has been established between EGFR expression levels and individual response to cetuximab [6]. Primary resistance to cetuximab is estimated to occur with a frequency of 13–35 % [4, 5]. Acquired resistance also appears more or less rapidly during the treatment, through mechanisms that are complex and to date only partially understood [7].

While every individual HNSCC bears a unique load of genomic alterations, therapeutic targeting is limited by the fact that the current level of genomic analysis does not translate into clear information regarding tumour sensitivity to most drugs [8]. New companion assays that would help to predict individual tumour sensitivity to cetuximab and other treatments of HNSCC would be of medical and economic interest. Such companion assays would help to personalize the medical treatment of patients with HNSCC, and would also be helpful for the optimization of phase II and III clinical trials of new targeted therapies. We and others have examined the possibility of cultivating fragments of human solid tumours for a short-period of time, and exposing them to targeted therapies in vitro [9–13]. Two preliminary studies in particular provide a proof of principle that short-term culture is applicable for exploring the heterogeneity of individual responses of HNSCC to cetuximab [12, 13]. In the present study, we prepared tumour slices from HNSCC samples and exposed them to a panel of targeted therapies.

## Methods

### Tumour samples

This study was conducted in compliance with the French legislation and the declaration of Helsinki regarding ethical principles for medical research involving human subjects. The use of surgically-resected solid tumours for research purposes in the laboratory of Biochemistry of the University Hospital of Amiens was approved by the Comité de Protection des Personnes Nord-Ouest (CPP NO ref. 2009/14). A written consent was obtained from the patients. No samples were obtained from any patients that were minor or physically or mentally unable to understand and give their consent to the use of surgical samples.

Head and neck tumour specimens were obtained from 18 adult patients who had been referred to the Head and Neck department of Amiens University Hospital (France) for a surgical procedure between September 2013 and September 2014. Surgery was the first line of treatment, without previous radiotherapy or chemotherapy. Different localisations and stages of squamous cell carcinoma from the upper aero-digestive tract are represented (Additional file 1: Table S1) [14].

### Preparation of tumour slices and culture

About 1 cubic cm of non-necrotic tumour was selected by the pathologist from each fresh surgical specimen. Tumour samples were prepared as 300 *μm* thick slices with a vibrating blade microtome (VT1200S Vibratome, Leica). Slices were maintained for 48 h in 24-well culture plates in Dulbecco’s Modified Eagle Medium (DMEM) culture medium, supplemented with 10 % fetal calf serum (PAN-Biotech), penicillin / streptomycin, and 1 % glutamine at 37 °C in a 5 % CO_2_ atmosphere.

### Drugs

Eight targeted therapies (cetuximab, sorafenib, erlotinib, tivaninib, masitinib, ponatinib, afatinib and rapamycin) were selected on the basis of their distinct reactivities toward oncogenic kinases (Table 1). Drug concentrations applied in the culture medium were chosen from previous pharmacological studies [7, 15–18]. Cetuximab was purchased from Merck-Serono. Sorafenib, erlotinib, tivaninib, masi tinib, ponatinib, afatinib were purchased by Euromedex (Souffelweyersheim, France). Rapamycin was purchased from Sigma (Sigma-Aldrich, France). Except for cetuximab, which was kept in saline, all other drugs were dissolved in DMSO and kept at – 20 °C before use.

**Table 1:** A summary of the drugs used in this study and their inhibitory spectrum against the cellular kinome

Name	Concentration used	Main kinase target	Reference
Cetuximab	30 μM	EGFR (Epidermal Growth Factor Receptor)	[7]
Erlotinib	1 μM	EGFR	[15]
Afatinib	10 μM	EGFR / ErbB2/ ErbB4	[15]
Sorafenib	10 μM	B-RAF, C-RAF, PDGFR-A, PDGFR-B (Platelet-Derived Growth Factor Receptor)	[15]
		VEGFR-2 (Vascular Endothelial Growth Factor-2)	
Masitinib	10 μM	KIT, PDGFR-A, PDGFR-B	[15]
Tivantinib	10 μM	HGFR (Hepatocyte Growth Factor Receptor)	[16]
Ponatinib	1 μM	FGFR1-4 (Fibroblast-Growth Factor Receptor), PDGFR-A, VEGFR-2	[17]
Rapamycin	1 μM	mTOR (mammalian Target Of Rapamycin)	[18]

### Immunohistochemistry

Tumour slices were fixed in formalin and then paraffin-embedded; 3 μm sections were cut and stained with hematoxylin phloxin saffron (HPS) to select non-necrotic and non-fibrotic areas with a high density of tumour cells. The monoclonal antibody MIB1 (Immunotech, Marseille, France) was used for the immunostaining of Ki67. Two tumour slides were analyzed for each experimental condition. Ten microscopic pictures were taken from each slide focusing on non-necrotic and non-fibrotic tumour areas, as defined by a senior pathologist (J.-F.I.). The cell proliferation index (CPI) was calculated from tumour cells only, excluding cells from the matrix and vessels. The ratio of Ki67-positive cells (brown stained nuclei / total number of nuclei × 100) was automatically determined by using ImmunoRatio, an Image J plugin adapted to automated image analysis (http://jvsmicroscope.uta.fi/sites/default/files/software/immunoratio-plugin/index.html). The total number of pictures analyzed was 20 per experimental condition, representing in total a minimum of 1000 tumour cells. Mean CPI was determined by calculating each time the average, using the results from all slides.

### Immunoblots

Tumour slices treated as indicated were kept at −20 °C for immunoblotting. Total extracts were prepared as described previously and loaded on polyacrylamide gels (SDS-PAGE) and transferred to nitrocellulose membranes [11]. Antibodies against Extracellular Regulated Kinase 1/2 (ERK) and phosphorylated ERK1/2 (pERK) were from Cell Signaling Technology (Danver, MA, USA). Antibodies against β actin were from Sigma (Saint-Quentin Fallavier France). Secondary antibodies coupled to peroxydase were from GE Healthcare (Aulnay-sous-Bois, France). Enhanced chemiluminescence reaction was used for revelation. Immunoblots were scanned and quantified using the software Image J (National Institute of Health, USA).

### Statistical analysis

The Student’s *t*-test was used for individual biological analysis and a value of *p < 0.05* was considered as threshold for significance. Pearson’s r test was used for correlation analysis. Hierarchical cluster analysis was performed with the software R3.02 (http://www.r-project.org/). The hclust function in R was used, using the agglomeration method “complete”. The algorithm proceeds iteratively. At each stage distances between clusters are recomputed by the Lance–Williams dissimilarity update formula.

## Results

### Short-term culture of tumour samples for the analysis of the effects of targeted therapies on individual HNSCC tumours

In order to analyse the impact of targeted therapies against HNSCC, we designed a panel consisting of eight targeted therapies that have reached at least phase II/III in clinical trials for the treatment of various solid tumours. This panel included the drugs cetuximab, sorafenib, erlotinib, tivaninib, masitinib, ponatinib, afatinib and rapamycin, and was selected on the basis of the distinct reactivities of these compounds against important oncogenic kinases (Table 1). Tumour samples were obtained from 18 patients addressed for surgical resection of HNSCC (Additional file 1: Table S1). Tumour slices were prepared from each tumour, and maintained for 48 h in culture with each anti-cancer drug applied at pharmacological concentrations. This time point was selected on the basis of our previous study showing a good preservation of cell viability and tumour architecture in these conditions [13]. Four out of the 18 tumour samples were not exploitable after 48 h of culture because there were not enough identifiable tumour cells identifiable at the time of histological examination. The reason for this failure to maintain these four tumours successfully in culture is not clear at this stage. Some possible explanations may include low tumour cellularity, pre-existence of necrotic areas, culture-induced necrosis, or a simultaneous occurrence of these.

**Fig. 1:**
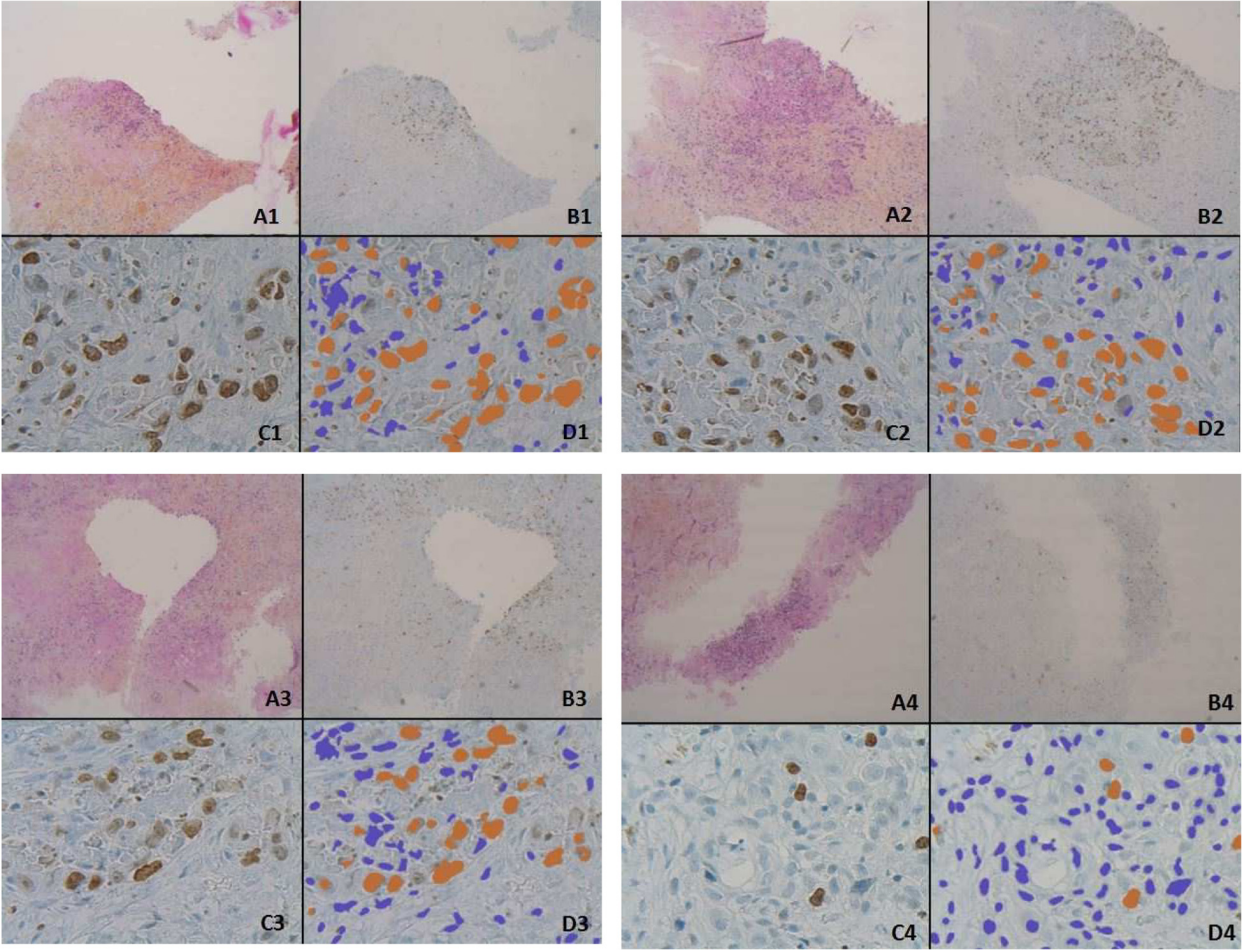
Representative microscopic acquisitions showing tumour cell proliferation assessed by Ki67 staining. Pictures are from tumour slices obtained from four different patients and analysed after 48 h of culture. Tumour slices were maintained in control conditions (**a**, **c**) or exposed to cetuximab (**b**, **d**). Ki67 immunostaining is shown in panels **a** and **b**. Automatic identification of stained nuclei is shown in panels **c** and **d**. The nuclei that are recognized as negative for Ki67-labelling are shown in blue, while those identified as positively stained are shown in orange. The four examples shown are representative of tumour areas with different proportions of proliferating cells, as defined by the % of Ki67 labelling (X 40 in **a**, **b**; X4 in **c**, **d**)

**Fig. 2:**
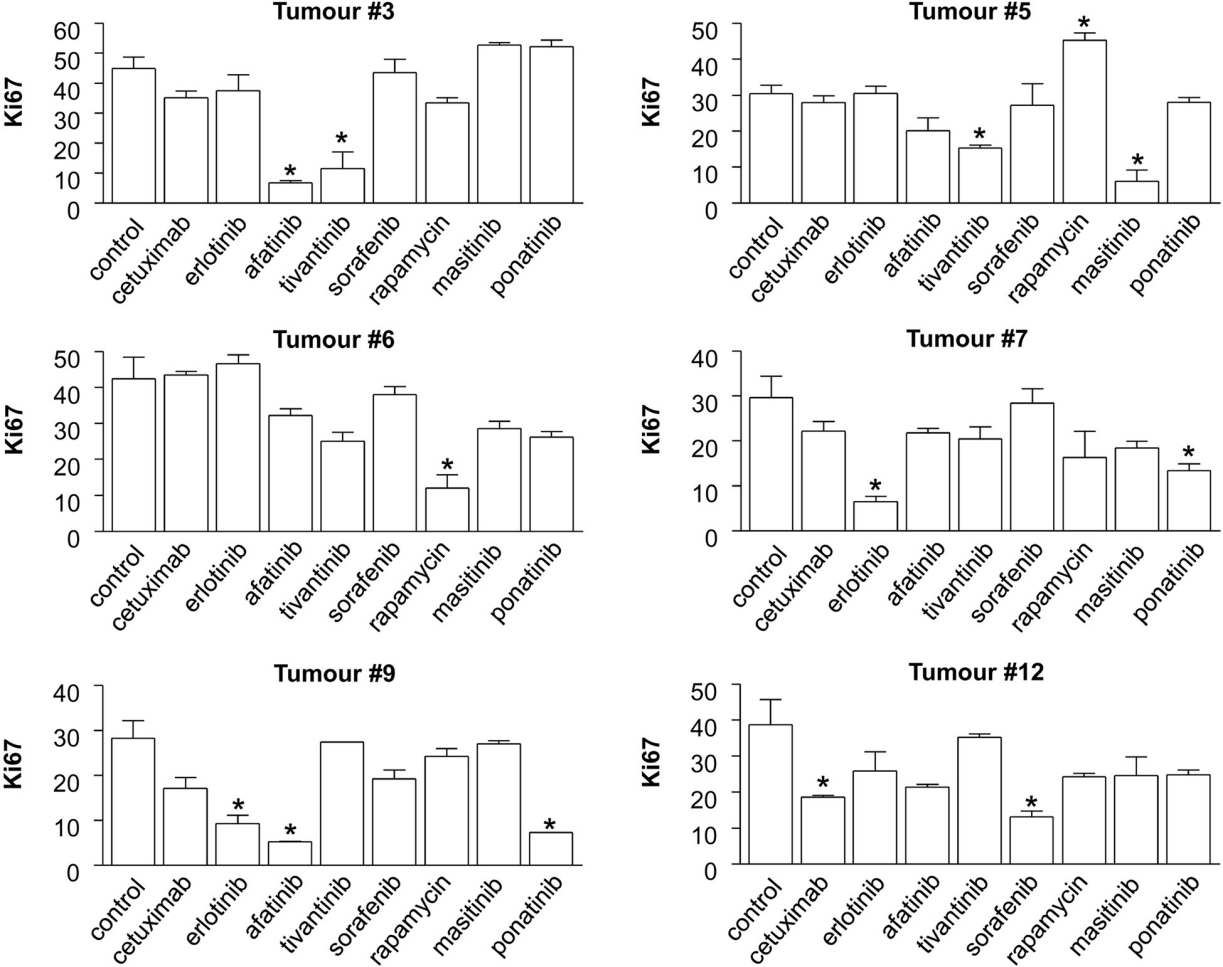
Effect of targeted therapies on the cell proliferation index in six representative HNSCC tumours in short-term culture. The histograms represent the average % of tumour nuclei stained with Ki67. The average presented is based on the automatic quantification of 20 tumour fields from two slides, representing a minimum of 1000 tumour cells for each condition, as indicated in the Materials and methods section. Each treatment was maintained in culture for 48 h. Note that each individual tumour presents a different % of Ki67-positive cells in control conditions, reflecting individual differences in the basal proliferative index. * indicates a statistically-significant difference compared to control conditions, corresponding to culture without treatment, with *p <0.05* (Student’s *t*-test)

We focused our analysis on the 14 other tumour samples. The effect of each treatment was analysed by exploring the % of tumour cells labelled with Ki67 by immunohistochemistry (Fig. 1). We found that the effect of treatment on cell proliferation greatly varied depending on the drug and the patient (Fig. 2). When we set the cut off for CPI inhibition induced by each drug in comparison to control at 50 %, cetuximab was shown to be active against two out of fourteen tumours. In some cases, the targeted therapy was shown to increase cell proliferation in comparison to control (i.e., rapamycin or tivantinib, in one tumour each). Sorafenib was the drug that was active in the largest proportion of tumours (5/14). Interestingly, ponatinib and masatinib were also active on some HNSCC tumours (3/14). In total, a more than 50 % CPI inhibition was observed in 10/14 tumours for at least one drug.

### Comparing the anti-proliferative efficacies of various therapeutic drugs using short-term culture of tumour slices

**Fig. 3:**
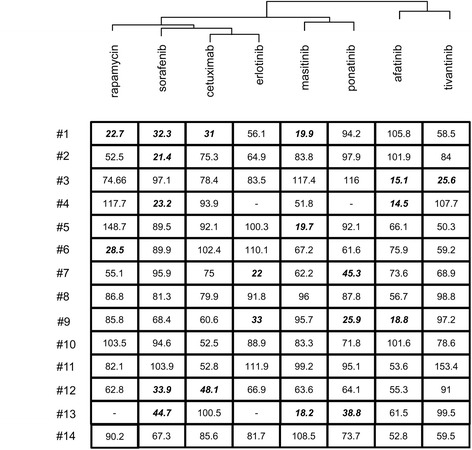
Dendrogram analysis exploring the anti-proliferative effects of targeted therapies in HNSCC slices. The analysis was conducted in the 14 HNSCC tumours, based on the calculated % inhibition of cell proliferation, i.e., based on the % of labelling of Ki67 in tumour cells, after normalization. Control condition for each tumour sample was taken as reference (100 %) for the calculation of the % inhibition of Ki67-labelling by each drug. This normalization step was applied in order to minimize the impact of the heterogeneous basal proliferative index of individual tumours, and in order to preferentially address the effects of the different drugs. Following this normalization, a cluster analysis was performed in order to identify drugs with neighbouring activities on Ki67 labelling. Bold and italic characters indicate conditions with a greater than 50 % inhibition of proliferation. Empty cases indicate conditions where tumour material was not sufficient for analysis

In order to explore the efficacy of each drug at the level of the entire tumour population, we performed a dendrogram analysis of the measurements obtained in the proliferation assays. The cluster analysis was performed using the values measured with Ki67 labelling (Fig. 3). However, compared to the data presented earlier, normalization step was introduced, with control condition taken as reference for the calculation of the % inhibition of Ki67-labelling by each drug. This normalization step was applied in order to minimize the impact of heterogeneous proliferative index in individual tumours and therefore facilitate the comparison of the effects of drugs. Interestingly, the subsequent cluster analysis revealed that some drugs displayed a shared pattern of efficacy with others (Fig. 3). The three drugs that were initially selected on the basis of their reactivity toward the EGFR-RAF-MEK-ERK pathway, i.e., cetuximab, erlotinib and sorafenib, displayed neighbouring activity in terms of effect on tumour cell proliferation in HNSCC. Interestingly, afatinib, another compound presumed to inhibit EGFR, did not cluster with cetuximab, erlotinib and sorafenib (Fig. 3). These findings suggest that afatinib did not exert its control over HNSCC tumour proliferation through the same mechanisms as the other three drugs.

### Exploring the impact of targeted therapies on the RAF-MEK-ERK cascade in individual tumours using short-term culture of tumour slices

**Fig. 4:**
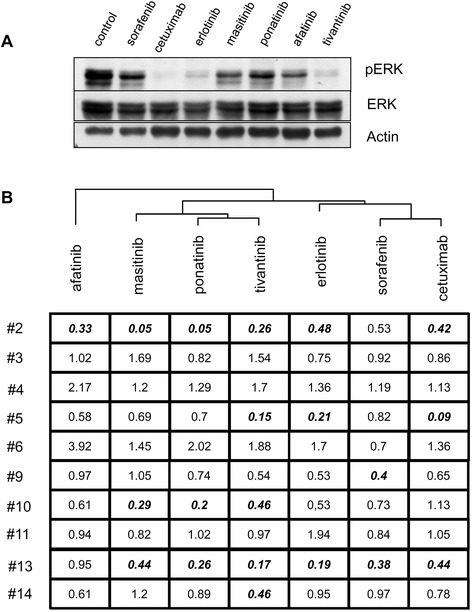
ERK1/2 kinase phosphorylation analysis for the exploration of the mode of action of targeted therapies against HSNCC. **a** Immunoblot analysis performed upon short-term culture of a representative HNSCC tumour*.* Sample fragments obtained from tumour #5 were processed and analysed by immunoblotting for the indicated markers, with β-actin used as a loading control. **b** Dendrogram analysis based on the pERK / ERK ratio calculated for each condition. For each condition, an immunoblot analysis was performed on one slice cultured as indicated. Normalization of pERK/ERK ratio was applied in order to minimize the impact of the heterogeneity of individual tumours, by setting the value of the pERK/ERK ratio to 1 for control conditions. Following this normalization, a cluster analysis was performed in order to identify drugs with neighbouring activities on ERK phosphorylation. Bold and italic characters indicate conditions with a greater than 50 % inhibition of ERK phosphorylation

In order to further explore the use of short-term culture of tumour fragments, an immunoblot analysis of the expression levels of the markers ERK and phospho-ERK (pERK) was performed on samples obtained in the same conditions as previously (Fig. 4). The levels of ERK phosphorylation, reflecting the activation of the RAF-MEK-ERK cascade, were analysed in 10 tumours for which sufficient tumour material was available. A representative immunoblot is shown in Fig. 4a. For all samples, a ratio of pERK/ERK was calculated after densitometric analysis of the immunoblot. This ratio was used for dendrogram analysis (Fig. 4b). Interestingly, cetuximab, erlotinib and sorafenib displayed neighbouring activity on the phosphorylation of ERK, while afatinib did not cluster with these three drugs (Fig. 4b). These findings were consistent with our previous analysis, centred on cell proliferation, and confirmed the relevance of the analysis of ERK phosphorylation in this setting.

**Fig. 5:**
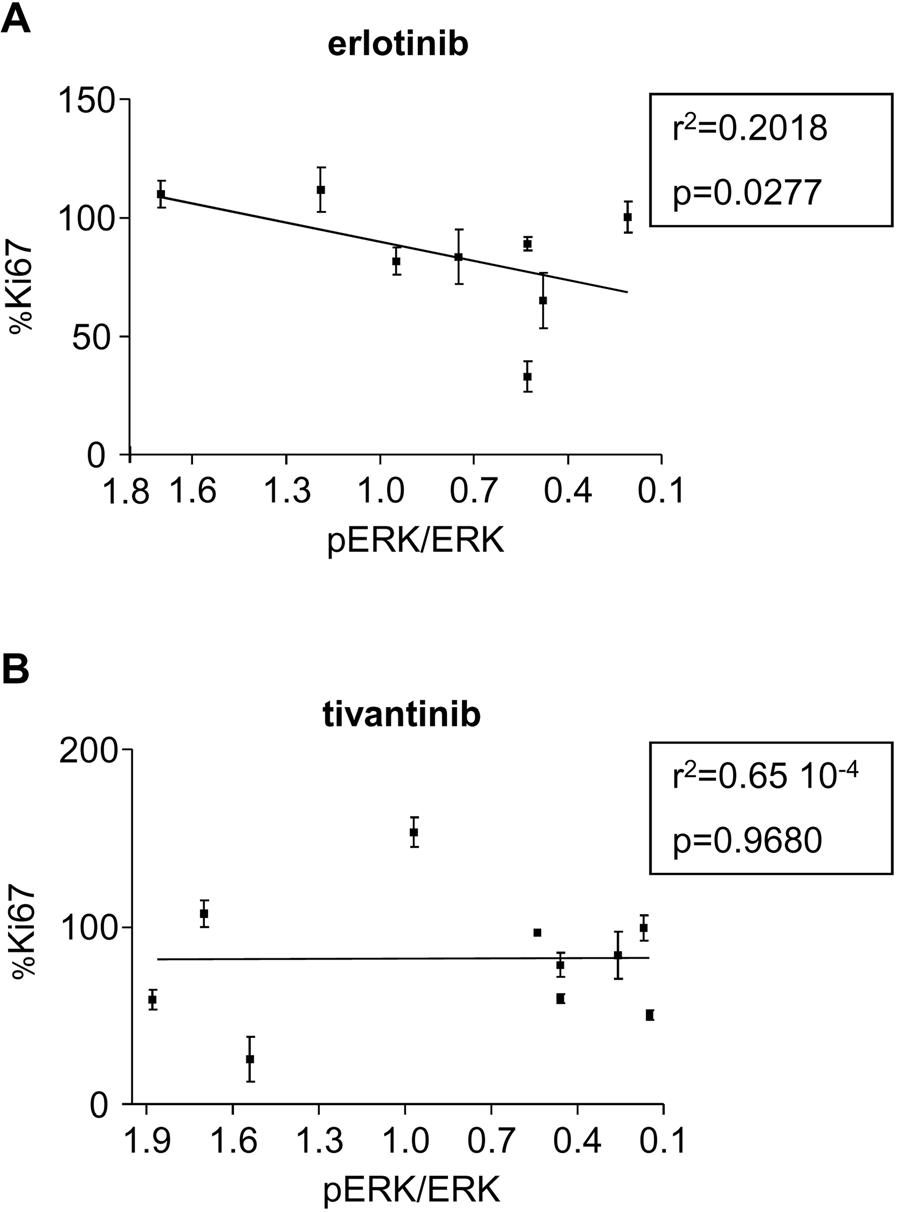
Correlation analysis exploring the mode of action of targeted therapies against HSNCC. A correlation is presented between the % reduction of Ki67 labelling induced by erlotinib (**a**) and tivantinib (**b**), and the % reduction of the pERK/ERK ratio. Each dot represents a single patient. Values of Pearson’s r test and the corresponding p value are presented each time

We further explored the possibility that the proliferation levels and pERK/ERK ratios might be linked by performing a correlation analysis for each drug (Fig. 5). Interestingly, a correlation was found between the control exerted over tumour cell proliferation and the RAF-MEK-ERK cascade for erlotinib (*r*^2^ = 0.2018, *p* = 0.0277 using Pearson’s r test) (Fig. 5a). No such correlation was found for tivantinib (*r*^2^ = 0.65 × 10^−4^, *p* = 0.9680) (Fig. 5b). We concluded that short-term culture of tumour slices is suitable for the exploration of the effects of therapeutic drugs on HNSCC.

## Discussion

Here, we explored the individual sensitivity of HNSCC tumours that were exposed in culture to various drugs, using a procedure that has been described by us and others and applied to a variety of human solid tumours in the past [9–13]. The tumour samples were sliced and maintained in culture for 48 h. This time point was chosen as a compromise, in order to let the tumour cells undergo in theory two proliferation cycles without inducing any massive cell death due to the cell culture conditions [13]. Following the identification of areas with high density of tumour cells, Ki67 labeling combined with automated image analysis was applied in order to examine the impact of a panel of targeted therapies on tumour cell proliferation. We show that short-term culture is a simple and robust method for the evaluation of the effects of targeted therapies on fresh human tumours, i.e., in conditions that resemble as closely as possible the clinical setting.

We observed that proliferation was inhibited by at least one drug in 10/14 HNSCC tumours in our experimental conditions. At this stage however, the aim of the present study was not to raise any definitive conclusions regarding the sensitivity of HNSCC to each of the drugs that was tested. Indeed, there are three major limitations to this study: i) the first limitation is the fact that a single pharmacological concentration of each drug was tested. Future studies should ideally assess a range of concentrations to determine an optimal inhibitory concentration for each drug. ii) The second major limitation of the use of tumour slices maintained ex vivo is that they reflect the consequences of the direct inhibition of signal transduction pathways inside tumour cells. Short-term culture of tumour slices cannot be used to evaluate anti-angiogenic or immunotherapeutic procedures, whose effects on tumour cells are indirect and require mid- to long-term exposure. ii) The third limitation of the present study is the limited number of tumours analysed (*n* = 14), which does not allow us to draw any definitive conclusions regarding the frequency with which tumours are resistant or sensitive to different drugs. Having in mind these limitations, it is nevertheless interesting to note that cetuximab, the only approved drug for the treatment of HNSCC that was tested in the panel, significantly inhibited the proliferation of only two tumour samples. An anti-proliferative effect was more often observed with sorafenib (*n* = 5), masitinib (*n* = 3), and afatinib (*n* = 3), thus opening the possibility that some HNSCC tumours might be individually sensitive to these drugs. At this stage, we conclude that short-term culture of tumour samples is a technique that could be useful for exploring the tumour-intrinsic determinants of drug sensitivity at the individual level.

Beyond this exploration of individual tumour sensitivity, cluster analysis that was built from the results of all drugs on tumour cell proliferation resulted in a pattern that was concordant with the known mechanisms of some of the drugs. Cetuximab, erlotinib and sorafenib, which are drugs that target the EGFR-RAF-MEK-ERK transduction pathway, presented related patterns of anti-proliferative efficacy. The observation validated the results of the assay, while providing another practical demonstration of the interest of short-term culture of tumour samples. Afatinib, whose mode of action is thought to be the inhibition of EGFR, was found to exhibit an activity spectrum distinct from those of the others EGFR-RAF inhibitors. In agreement with these observations, a correlation was established between the extent of the inhibition of ERK phosphorylation and the control of HNSCC proliferation with erlotinib, but not with afatinib. Given the poor specificity of most inhibitors of tyrosine kinase currently in clinical use, additional mechanisms of action beyond EGFR inhibition and the control of the EGFR-RAF axis can therefore be hypothesized for a drug like afatinib [15]. On the basis of these data, we suggest that short-term culture of tumour samples can also be useful for addressing the mode of action of anticancer drugs on HNSCC.

Compared to other techniques aimed at exploring the sensitivity of individual solid tumours in general and HNSCC in particular, the preparation of slices offers the advantage of being relatively easily performed and rapidly implemented. It might be therefore suited for rapid exploration of the response of a relatively-large number of tumours exposed to anticancer drugs. In theory, we propose that short-term culture of slices might serve as a basis for new companion assays that might be useful for the design of clinical trials testing the efficacy of anti-cancer drugs [19]. Ultimately, it is also tempting to propose that it could be used to select the most active targeted therapies for individual patients with advanced HNSCC. More studies are however required in order to establish the optimal culture conditions, as well as the biochemical and histological readouts that best reflect the efficacy of anticancer drugs against HNSCC. Validation of the short-term culture assay will require a comparison with the results obtained with other, more established models currently used for the determination of tumour drug-sensitivity, such as patient-derived xenografts [18]. Finally, in the present study, we did not carry out a correlation of the in vitro behaviour of tumour cells with the clinical response, since most patients enrolled here were considered cured after surgery and thus did not receive any targeted therapy.

## Conclusion

Short-term culture of tumour slices can be applied to evaluate the effects of targeted therapies on freshly-resected human HNSCC tumours. Future in vitro studies and clinical trials are required in order to: i) optimize the conditions for the assessment of tumour sensitivity in individual patients; ii) compare the results of the assay with the clinical response of patients with advanced / metastatic HNSCC. Together with other functional assays that explore complex aspects of cancer cell physiology, such as the functional state of the cell death machinery [20] and genomic assays, short-term culture of tumour fragments may offer new possibilities for the optimisation of treatment for individual cancer patients.

## Additional file

Additional file 1: Tables S1 A summary of the clinical characteristics of the patients. (DOC 57 kb)

## Abbreviations

CPI: cell proliferation index
EGFR: epidermal growth factor receptor
ERK: extracellular-signal-regulated kinases
HNSCC: head and neck squamous cell carcinomas
HPS: hematoxylin phloxin saffron
