# Supplementary material for: Short-term culture of tumour slices reveals the heterogeneous sensitivity of human head and neck squamous cell carcinoma to targeted therapies
Source: BMC Cancer. 2016 Apr 16;16:273. doi: 10.1186/s12885-016-2318-x (PMC4834185; doi:10.1186/s12885-016-2318-x)
Supplement: Additional file 1: Tables S1 — A summary of the clinical characteristics of the patients. (DOC 57 kb) [file 12885_2016_2318_MOESM1_ESM.doc]

Suppl. Table 1: A summary of the clinical characteristics of the patients.

Abbreviations used are: SCC: Squamous Cell Carcinoma, WD: Well differentiated, MD: Moderately differentiated, PI: Perineural invasion, VI: Vascular invasion. The type of Squamous Cell Carcinoma (SCC) refers to the World Health Organization (WHO) classification of Head and Neck Tumours [14].

| **Patient** | **Sex** | **Age** | **Organ** | **TNN** | **Type of SCC** | **Macroscopic examination** | | | **Microscopic examination** | | |
| --- | --- | --- | --- | --- | --- | --- | --- | --- | --- | --- | --- |
|  |  |  |  |  |  | **Infiltrative** | **Ulcerative** | **Budding** | **Grade** | **P I** | **V I** |
| 1 | **Female** | **59** | Tongue | T2N0M0 | Conventional | * |  |  | WD | * |  |
| 2 | **Male** | **53** | Piriform Sinus | T3N2bM0 | Conventional | * |  |  | WD | * |  |
| 3 | **Male** | **59** | Larynx | T4N2cM0 | Conventional | * |  | * | WD |  |  |
| 4 | **Male** | **48** | Valleculae | T2N0M0 | Conventional | * | * | * | WD |  |  |
| 5 | **Male** | **60** | Larynx | T4N1M0 | Conventional | * |  | * | WD |  |  |
| 6 | **Female** | **66** | Oral cavity | T2N0M0 | Conventional | * |  |  | WD |  |  |
| 7 | **Male** | **47** | Tonsillolingual sulcus | T2N2bM0 | Conventional | * |  |  | MD |  |  |
| 8 | **Male** | **53** | Pelvilingual sulcus | T2N3M0 | Conventional | * |  |  | MD |  |  |
| 9 | **Male** | **74** | Oral cavity | T2N0M0 | Conventional | * | * |  | WD |  |  |
| 10 | **Male** | **63** | Palatine tonsil | T4N2bM0 | Conventional | * |  |  | WD |  |  |
| 11 | **Male** | **57** | Base of tongue | T4N0M0 | Conventional | * |  |  | WD | * | * |
| 12 | **Male** | **56** | Larynx | T3N2cM0 | Conventional | * |  |  | WD |  |  |
| 13 | **Male** | **57** | Retromolar trigone | T4N0M0 | Conventional | * | * |  | WD |  |  |
| 14 | **Male** | **54** | Palatine tonsil | T1N1M0 | Conventional | * | * |  | WD |  |  |
